# Macromineral and trace element requirements for Santa Ines sheep

**DOI:** 10.1038/s41598-021-91406-w

**Published:** 2021-06-10

**Authors:** Dayanne Lima Sousa, Marcos Inácio Marcondes, Luciano Pinheiro Silva, Francisco Wellington Rodrigues Lima, Caio Julio Lima Herbster, Jocely Gomes Souza, João Paulo Pacheco Rodrigues, Leilson Rocha Bezerra, Ronaldo Lopes Oliveira, Elzania Sales Pereira

**Affiliations:** 1 Department of Animal Science, Federal University of Ceara, Mister Hull Avenue, Fortaleza, 297760356000 Brazil; 2 Department of Animal Science, Washington State University, Pullman, WA 99164 USA; 3 Institute of Studies of Humid Tropics, Federal University of Southern and Southeastern Para, Xinguara, 68557335 Brazil; 4 Department of Animal Science, Federal University of Campina Grande, Universitaria Avenue, Patos, Paraiba 58708110 Brazil; 5 Veterinary Medicine and Animal Science Department, Federal University of Bahia, 500 Adhemar de Barros Avenue, Salvador, 40170110 Brazil

**Keywords:** Metabolism, Animal physiology

## Abstract

Minerals play an important role in animal metabolism. Knowledge of mineral requirements allows well-formulated diets to be provided, which is the main factor that affects performance. To determine the macromineral and trace element requirements for growth and maintenance, thirty-eight 2-month-old Santa Ines lambs with initial body weight (BW) of 13.0 ± 1.49 kg were distributed in a factorial design with feeding levels (ad libitum*,* 30% and 60% feed restriction) and sex classes [castrated (CM) and intact males (IM)]. The net mineral requirements for gain were higher (*P* < 0.05) with increasing BW and average daily gain, except for Ca and Na, which remained constant as the empty BW (EBW) increased. The macromineral net requirement for maintenance (g/kg EBW^0.75^) and the true retention coefficient (k; %) were 0.0784 and 65.2 for Ca, 0.0926 and 80.0 for P, and 0.0379 and 59.0 for K, respectively. The k of Mg was higher (*P* < 0.05) for IM (11.3 for IM and 7.9 for CM). Sex did not affect (*P* > 0.05) the maintenance requirement of the trace elements Co, Cu, Zn and Cr which were 0.0015, 0.037, 0.698, and 0.0055 (mg/kg EBW^0.75^), respectively. Our study indicated that the Santa Ines net mineral requirements are different from the main nutritional requirements established by committees for sheep, which may result in unbalanced diets.

## Introduction

The determination of mineral requirements of small ruminants is a key step to properly formulate and balance their diets, especially for animals in a growing phase. In Brazil, ruminant diets are formulated according to the recommendations proposed by the ARC^1^, NRC^2,3^, AFRC^4^, CSIRO^5^ and INRA^6^ committees. In general, the dataset of those committees is based on data obtained from trials using wool sheep. However, it is unknown whether the requirements of hair sheep in the tropics have been carefully considered, especially given their phenotypic differences, potential for weight gain, and body composition, which are known to affect the nutritional requirements of animals^7^.

Mineral requirements are generally based on supplementation^3^, which disregards the mineral composition of the feed. The mineral requirement knowledge has a global importance. Among the hair sheep breeds that are well adapted to environmental conditions in warm areas, Santa Ines sheep are a dominant breed for meat production. The amount of mineral retained in the animal's body in relation to that consumed is necessary to determine the need for macrominerals and trace elements for the maintenance and growth of the animals^8^ as well as to decrease unnecessary excretion of those minerals in the environment^9^.

The majority of feeding systems for sheep^3,5,10^ use the absorption coefficient of each mineral to calculate dietary requirements because they consider that the excretion of minerals through the urine is minimal^1^. The maintenance requirement for minerals is determined from the knowledge of the minerals retained in the animal's body in relation to the minerals consumed^8^. One of the advantages of using the retention coefficient is that it indicates the real relationship between consumed and retained minerals in the animal's body.

We hypothesized that the demand for minerals by Santa Ines hair sheep may be different from wool sheep due to the peculiarities of these animal. The objective of this study was to evaluate the body composition and the macromineral and trace element requirements for Santa Ines sheep.

## Methods

### Local, animal studies and data statement

This study was conducted at the Animal Nutrition Laboratory of the Department of Animal Science of the Federal University of Ceara in Fortaleza, Ceara State, Brazil (30° 43′ 02″ S, 33° 32′ 35″ W). Animal experiments were conducted in accordance with the Guiding Principles for the Care and Use of Research Animals developed by the Federal University of Ceara, Fortaleza, Brazil. The protocol and methods were approved by the Ethics Committee on Animal Research of the Federal University of Ceara (No. 98/2015). Information provided in the manuscript complies with the essential recommendations for reporting of the ARRIVE guidelines.

### Animals, diets, and experimental design

Thirty-eight 60-day-old Santa Ines hair lambs averaging 13.0 ± 1.49 kg body weight (BW) were allocated in individual stalls. The sheep were dewormed using 0.3 mL of ivermectin (Ivomec; Merial, Duluth, GA, USA), vaccinated for clostridiosis using 4 mL of polyvalent *Clostridium* vaccine per animal (Poli-star; Vallée, Montes Claros, Brazil), and later supplemented with 0.3 mL of vitamins ADE (Vit A, 20.000.000 IU; Vit D3, 5,000,000 IU; and Vit E, 6000 IU/100 mL; Vallée, Montes Claros, Brazil). Among the 38 sheep, 19 were chosen at random and subjected to castration using the Burdizzo method of castration. In brief, 2.5–5 mL of local anesthetic in 2% xylocaine was injected subcutaneously on each side of the spermatic cord, and the operation was performed after 5 min. The animals were submitted to an adaptive period of 15 days. Posteriorly, four lambs of each sex [4 castrated male (CM) and 4 intact male (IM) Santa Ines lambs] were randomly selected and slaughtered to serve as a baseline group (Table S1). Then, the remaining lambs (n = 30) were assigned to a completely randomized design with three feeding levels (ad libitum*,* 30% and 60% feed restriction) and two sexes (CM and IM) composing a 3 × 2 factorial scheme. The experimental rations were formulated to meet a gain of 200 g/day as recommended by the NRC^3^.

The lamb diet consisted of 600 g/kg hay and 400 g/kg concentrate (Table 1). Feed was offered as a total mixed ration (TMR) two times per day (at 08:00 and 16:00 h). The orts from the lambs in the ad libitum group were removed before each morning feeding and weighed to calculate the intake and feeding level of the lambs undergoing 30% and 60% feed restriction. Water was provided ad libitum for all animals. The animals were weighed every seven days to calculate body weight gain (BWG). All lambs were slaughtered when the lambs in the ad libitum group reached an average of 30 kg BW. Based on that criterion, the experimental trial lasted 100 days.

**Table 1:** Chemical composition (%) of feedstuffs and experimental diet.

Item	Total ration^a^	Tifton 85 hay	Corn ground	Soybean meal
**%DM**				
Dry matter	91.81	92.95	90.05	90.12
Crude protein	17.80	11.06	7.70	50.01
Ether extract	2.47	1.53	6.63	1.18
Ash	5.67	6.69	1.50	7.03
NDF	49.2	70.70	15.29	19.38
NDFap	45.64	67.35	14.12	12.80
**Macromineral (%DM)**				
Calcium	0.16	0.30	45.02	0.32
Phosphorus	0.17	0.58	5.63	0.16
Magnesium	0.41	0.54	12.86	0.28
Sodium	0.16	0.17	1.84	0.16
Potassium	8.82	5.05	2.73	4.92
**Trace mineral (%DM)**				
Cobalt	0.03	0.09	0.03	0.04
Copper	0.09	0.09	0.03	0.04
Manganese	0.03	0.03	0.09	0.02
Zinc	0.02	0.09	0.04	0.01
Chrome	0.09	0.09	0.09	0.09

*DM* dry matter, *NDFap* neutral detergent fiber corrected for ash and protein, *NDF* neutral detergent fiber.

^a^Total ration with ingredient proportions of the total ration (g/kg as fed): Tifton 85 hay (60%); ground corn (20.07%); soybean meal (19.23%); limestone (0.19%); dicalcium phosphate (0.41%); sodium chloride (0.07%) and mineral premix (0.03%). Composition of mineral premix (1 kg of premix): calcium, 225 g to 215 g; phosphorus, 40 g; sulfur, 15 g; sodium, 50 g; magnesium, 10 g; cobalt, 11 mg; iodine, 34 mg; manganese, 1800 mg; selenium, 10 mg; zinc, 2000 mg; iron, 1250 mg; copper, 120 mg; fluor, 400 mg; vitamin A, 37.5 mg; vitamin D3, 0.5 mg; and vitamin E, 800 mg.

### Slaughter procedures

Slaughter procedures were carried out in accordance with regulatory standards established by the Animal Care and Use Committee. Before slaughter, the animals were deprived of solid and liquid feed for 18 h. The animals were then stunned per a brain concussion and slaughtered by cutting the jugular vein. All non-carcass components (liver, heart, lungs, trachea, tongue, kidneys, spleen, hide, head, and feet) and the digestive tract (rumen, reticulum, omasum, abomasum, small intestines, and large intestines) were weighed, emptied, cleaned, and weighed again.

Blood was weighed and mixed with the other body parts to be further chemically analyzed. All body parts were weighed separately, frozen, ground, and mixed to be later chemically analyzed. Measurement of body mass was obtained by determining only the empty BW (EBW), which was obtained after the animal was slaughtered. The EBW was calculated as the difference between the BW at slaughter and the contents of the gastrointestinal tract and bladder.

Subsequently, the carcasses were refrigerated at 4 °C for 24 h. Posteriorly, the carcasses were divided in half (right and left carcasses). The right half-carcass and all non-carcass components were frozen, subsequently sawed with a band saw, and ground with an industrial cutter. After homogenization, 500 g of carcass and non-carcass components was sampled and stored in a freezer at − 20 °C.

### Chemical analyses

For performing chemical analyses, 500 g of each sample of the organ mass, half-carcass mass of the ground organs, blood, hooves, head, and right half of the carcass and hide were dried in a forced-ventilation oven at 55 °C for 72 h. Posteriorly, the samples were defatted by extraction with ether in a Soxhlet apparatus (Association of Official Analytical Chemists (AOAC)^11^; method number 920.39) and then ground in a ball mill. Dry matter (DM) was determined by using the method 967.03 from the AOAC^11^. The ash (AOAC, 1990; method 942.05)^11^ and crude protein (AOAC, 1990; method 981.10)^11^ levels were determined from fat-free samples. The body water content was determined as 100% minus the DM.

Samples of feed, body, rations, and orts were evaluated for mineral composition through digestion in nitroperchloric acid, according to the INCT-CA M-004/1 method described by Detmann et al.^12^, thus obtaining a mineral solution from which dilutions were made to quantify the minerals. The Ca and Mg concentrations were determined by adding strontium chloride and using atomic absorption spectrometry (GBC Avanta Sigma, Hampshire, USA) (Method 968.08; AOAC)^13^. The Na and K concentrations were determined by flame emission spectrometry (Corning 400, NY, USA) (Method 985.35, AOAC)^13^. The P concentration was determined by reduction of the phosphorus-molybdate complex with ascorbic acid followed by measurement using a colorimetric spectrophotometer (Method 965.17, AOAC)^13^. The Co, Cu, Mn, Zn, and Cr concentrations were determined by spectrometry (GBC Avanta Sigma, Hampshire, USA) (Method 968.08; AOAC)^13^.

### Models and calculations

The retained mineral content was determined by the difference in the amount of minerals in the initial EBW (reference animals) and final EBW (experimental animals).

The model of Lofgreen and Garrett^14^ was used to estimate the net requirements for maintenance according to the following equation:

1 $${\text{RM}} = a + b \times {\text{MI}}$$

where RM is the retained mineral (mg kg EBW^0.75^ day); MI is the mineral intake (mg kg EBW^0.75^ day); *a* is the maintenance requirements; and *b* is the retention coefficient.

The model of Brody^15^ was used to estimate the body composition of minerals in the EBW as follows:

2 $${\text{B}}CM = a \times {\text{EBW}}^{b}$$

where BCM is the body content of the mineral (g); and ‘a’ and ‘b’ are regression parameters.

The net requirements of the minerals for gain were estimated by deriving Eq. (2) as follows:

3 $${\text{NMg}} = {\text{EBWG}} \times \left( {a \times {\text{b}} \times {\text{EBW}}^{{({\text{b}} - 1)}} } \right)$$

where NMg is net requirement for weight gain (g/day); ‘a’ and ‘b’ are parameters of the equation; and EBW is expressed in kilograms.

The requirements based on the EBW were converted to the BW using 1.18, a factor of conversion derived from the relationship between BW and EBW.

### Statistical analyses

Significance of treatments was verified by a two-way ANOVA model as follows: Yij = μ + Si + Rj + (SR)ij + eij; where Yij is the response variable, i.e., body composition parameters; Si the effect of sex (CM or IM); Rij is the feed restriction (ad libitum, 30% and 60% feed restriction); (SR)ij is the interaction of feed restriction by sex; and eij is the term for residuals.

Net requirements for maintenance and true retention coefficient were estimated using Eq. (1) through mixed models and testing sexual class effects using Eqs. (1) and (2). If parameters differed from zero, a variance component structure for the (co)variance matrix was used to fit equations. The Gauss–Newton method was used to solve nonlinear models for growth, and a P-value threshold of 0.05 was adopted to consider parameter differences. Influential records were not used when studentized residuals were greater than an absolute value of two.

All analyses were performed by SAS software (SAS Inst. Inc., Cary, NC) using the GLM procedure with a level of significance equal to 0.05 for two-way ANOVA and NLIN for nonlinear models.

## Results

### Dry matter (DM) intake, mineral intake, and body parameters

The DM and mineral intake decreased with increasing dietary restriction (*P* < 0.05; Table 2), but there was no sex effect (*P* > 0.05). The Na content in the EBW was higher in animals with 60% feed restriction (*P* = 0.001) than in the other animals.

**Table 2:** Dry matter and mineral intake of intact and castrated male Santa Ines hair sheep.

Item	Feeding restriction (FR)			Sex (S)^d^		*P* value			
	ad libitum	30%	60%	IM	CM	SEM	FR × S	FR	S
DMI (g/day)	811.2^a^	578.5^b^	330.2^c^	575.4	571.2	24.28	0.718	0.001	0.818
**Macromineral (g/day)**									
Ca	3.57^a^	2.33^b^	1.33^c^	2.43	2.39	0.10	0.951	0.001	0.599
P	2.90^a^	1.84^b^	1.05^c^	1.93	1.92	0.08	0.733	0.001	0.872
Mg	1.66^a^	1.13^b^	0.65^c^	1.16	1.14	0.04	0.917	0.001	0.571
Na	4.53^a^	3.19^b^	1.82^c^	3.20	3.16	0.13	0.871	0.001	0.682
K	10.88^a^	8.40^b^	4.80^c^	8.14	7.91	0.32	0.961	0.001	0.378
**Trace mineral (mg/day)**									
Co	2.69^a^	1.80^b^	1.02^c^	1.84	1.83	0.07	0.552	0.001	0.897
Cu	8.29^a^	6.20^b^	3.54^c^	6.05	5.98	0.26	0.851	0.001	0.745
Mn	47.27^a^	27.59^b^	15.75^c^	30.34	30.06	1.20	0.838	0.001	0.778
Zn	33.03^a^	24.07^b^	13.74^c^	23.74	23.48	0.95	0.814	0.001	0.748
Cr	0.36^a^	0.32^a^	0.18^b^	0.30	0.27	0.04	0.715	0.001	0.403

^d^*IM* intact male, *CM* castrated male, *DMI* dry matter intake.

Different letters (^abc^) indicate that the feeding restriction differed (*P* < 0.05; Tukey–Kramer test).

The Mg (0.56 vs. 0.68), Na (1.41 vs. 1.59), Co (1.49 vs. 1.64), and Mn (1.18 vs. 1.40) concentrations were higher (*P* < 0.05) to CM than IM whereas the other minerals were similar between the sexes (Table 3). The equations for estimating body composition and net gain requirements are shown in Table 4.

**Table 3:** Body parameters and mineral composition in the EBW of intact and castrated male Santa Ines hair sheep.

Item	Feeding restriction (FR)			Sex (S)^d^			*P* value		
	ad libitum	30%	60%	IM	CM	SEM	FR × S	FR	S
BWi (kg)	14.33	14.43	14.53	14.46	14.40	0.65	0.988	0.955	0.923
BWf (kg)	30.13^a^	23.87^b^	17.27^c^	24.20	23.31	0.55	0.556	0.001	0.065
EBW (kg)	23.00^a^	17.70^b^	12.15^c^	17.92	17.32	0.42	0.381	0.001	0.106
ADG (g/day)	155.2^a^	91.0^b^	26.7^c^	95.3	86.6	0.007	0.674	0.001	0.150
**Macromineral (g/kg EBW)**									
Ca	10.55	11.84	11.77	11.18	11.59	0.76	0.416	0.206	0.522
P	8.60	8.83	9.27	8.94	8.86	0.39	0.677	0.251	0.811
Mg	0.62	0.60	0.64	0.56^B^	0.68^A^	0.05	0.303	0.7307	0.004
Na	1.44^b^	1.50^ab^	1.56^a^	1.41^B^	1.59^A^	0.03	0.407	0.001	0.001
K	2.09	2.22	2.15	2.21	2.10	0.07	0.346	0.213	0.059
**Trace mineral (mg/kg EBW)**									
Co	1.54	1.54	1.62	1.49^B^	1.64^A^	0.07	0.107	0.495	0.023
Cu	6.40	7.36	7.04	6.45	7.41	0.85	0.676	0.547	0.188
Mn	1.30	1.17	1.38	1.18^B^	1.40^A^	0.10	0.154	0.138	0.015
Zn	41.36	36.98	37.36	38.55	38.58	1.99	0.320	0.083	0.984
Cr	2.69	2.17	2.57	2.40	2.55	0.40	0.388	0.419	0.665

^d^*IM* intact male, *CM* castrated male, *BWi* initial body weight, *BWf* final body weight, *EBW* empty body weight, *ADG* average daily gain.

Different letters (^abc^) indicate that the feeding restriction differed (*P* < 0.05; Tukey–Kramer test).

Different letters (^AB^) indicate that the sex differed (*P* < 0.05; Tukey–Kramer test).

**Table 4:** Equations for estimating body composition and net gain requirements of intact and castrated male Santa Ines hair sheep.

Item	Allometric equation	R^2^	RMSE	Equations
EBW (kg)	EBW = − 1.0887 + 0.9575 × BW^a^	0.98	0.228	–
EBWG (kg/day)	EBWG = − 0.0014 + 0.8199 × ADG^a^	0.98	0.007	–
**Macromineral (g)**				
Ca	Ca = 12.94 × EBW^0.99^	0.91	18.20	Ca = EBWG × (12.81 × EBW^−0.01^)
P	P = 8.57 × EBW^1.05^	0.91	15.77	P = EBWG × (8.99 × EBW^0.05^)
Mg^b^	Mg^b^ = 0.37 × EBW^1.17^	0.84	1.97	Mg^b^ = EBWG × (0.43 × EBW^0.16^)
Mg^c^	Mg^c^ = 0.37 × EBW^1.27^			Mg^c^ = EBWG × (0.46 × EBW^0.27^)
Na	Na = 1.64 × EBW^1.01^	0.94	2.22	Na = EBWG × (1.65 × EBW^0.01^)
K	K = 1.86 × EBW^1.10^	0.93	3.52	K = EBWG × (2.04 × EBW^0.10^)
**Trace element (mg)**				
Co	Co = 0.12 × EBW^1.09^	0.88	3.29	Co = EBWG × (0.13 × EBW^0.09^)
Cu	Cu = 3.80 × EBW^1.21^	0.67	29.10	Cu = EBWG × (4.59 × EBW^0.21^)
Mn^b^	Mn^b^ = 0.60 × EBW^1.21^	0.83	4.20	Mn^b^ = EBWG × (0.72 × EBW^0.21^)
Mn^c^	Mn^c^ = 0.60 × EBW^1.32^			Mn^c^ = EBWG × (0.79 × EBW^0.32^)
Zn	Zn = 16.21 × EBW^1.31^	0.93	77.42	Zn = EBWG × (21.23 × EBW^0.31^)
Cr^b^	Cr^b^ = 0.37 × EBW^1.61^	0.63	230.88	Cr^b^ = EBWG × (0.59 × EBW^0.61^)
Cr^c^	Cr^c^ = 0.37 × EBW^1.69^			Cr^c^ = EBWG × (0.62 × EBW^0.69^)

*BW* body weight, *EBW* empty BW, *EBWG* empty BW gain, *ADG* average daily gain, *RMSE* root mean square error.

^a^According to Pereira et al.^9^; ^b^intact male; ^c^castrated male.

### Net mineral and trace element requirements

Sex affected (*P* < 0.05) the concentrations of Mg, Mn, and Cr in the empty BW gain (EBWG; Table 4). The net mineral requirements for gain were higher (*P* < 0.05) with increasing BW and average daily gain (ADG), except for Ca and Na, which remained constant as the EBW increased (Table 5).

**Table 5:** Net mineral requirements for gain and total dietary requirements of intact and castrated male Santa Ines hair sheep.

BW (kg)	ADG (g/day)	Ca	P	Mg^a^	Mg^b^	Na^a^	Na^b^	K
**Net requirements for gain (g/day)**								
10	100	0.86	0.68	0.04	0.06	0.12	0.12	0.17
	200	1.73	1.38	0.08	0.11	0.23	0.23	0.35
20	100	0.85	0.71	0.05	0.07	0.12	0.12	0.19
	200	1.71	1.43	0.09	0.14	0.23	0.23	0.38
30	100	0.85	0.72	0.05	0.08	0.12	0.12	0.19
	200	1.71	1.46	0.10	0.16	0.24	0.24	0.39
**Dietary requirements (g/day)**								
10	100	1.68	1.23	0.44	0.81	0.19	0.17	0.44
	200	3.01	2.10	0.81	1.53	0.33	0.31	0.74
20	100	2.08	1.69	0.57	1.10	0.26	0.21	0.64
	200	3.41	2.59	0.99	1.98	0.40	0.35	0.96
30	100	2.49	2.13	0.69	1.33	0.32	0.25	0.83
	200	3.81	3.05	1.14	2.32	0.47	0.39	1.16

*BW* body weight, *EBW* empty BW, *ADG* average daily gain.

^a^Intact male; ^b^castrated male.

The net requirements of Mg and Mn for gain were 35% higher for CM than for IM with 30 kg BW and an ADG of 200 g (Tables 5, 6). The dietary requirements of Mg and Mn were 50.8% and 29.3% higher, respectively, for IM than for CM.

**Table 6:** Net trace mineral requirements for gain and total dietary requirements of intact and castrated male Santa Ines hair sheep.

BW (kg)	ADG (g/day)	Co	Cu	Mn^a^	Mn^b^	Zn	Cr^a^	Cr^b^
**Net requirements for gain (mg/day)**								
10	100	0.011	0.49	0.08	0.11	2.81	0.15	0.19
	200	0.022	0.99	0.16	0.22	5.68	0.30	0.37
20	100	0.012	0.58	0.09	0.14	3.56	0.24	0.31
	200	0.023	1.16	0.18	0.27	7.17	0.47	0.63
30	100	0.012	0.63	0.10	0.16	4.06	0.31	0.42
	200	0.024	1.27	0.20	0.31	8.18	0.62	0.84
**Dietary requirements (mg/day)**								
10	100	0.09	3.63	25.19	32.67	20.81	0.19	0.23
	200	0.17	6.77	44.79	59.87	29.01	0.38	0.47
20	100	0.10	4.79	35.20	46.67	37.33	0.31	0.40
	200	0.20	8.47	58.16	81.32	47.70	0.60	0.80
30	100	0.12	5.75	44.00	58.33	53.16	0.40	0.54
	200	0.21	9.77	69.11	98.03	64.99	0.79	1.07

*BW* body weight, *EBW* empty body weight, *ADG* average daily gain.

^a^Intact male; ^b^castrated male.

The equations used to estimate the requirements of macrominerals and trace elements for maintenance are shown in Table 7. The macromineral net requirement for maintenance (g/kg EBW^0.75^) and the true retention coefficient (k; %) were 0.0784 and 65.2 for Ca, 0.0926 and 80.0 for P, and 0.0379 and 59.0 for K, respectively. The Mg requirement was similar between sexes (0.0065), but the true retention coefficient was different (11.3 for IM and 7.9 for CM). The Na maintenance requirement was different between the sexes (0.0242 vs. 0.0167 for IM and CM, respectively), but the k (84.3) was similar. Sex did not affect (*P* > 0.05) the maintenance requirement of the trace elements Co, Cu, Zn and Cr which were 0.0015, 0.037, 0.698, and 0.0055 (mg/kg EBW^0.75^), respectively.

**Table 7:** Regression equations of the minerals retained from mineral intake to estimate the net maintenance requirements for macrominerals and trace elements of intact and castrated male Santa Ines hair sheep.

Item	Equations	R^2^	RMSE	*P* value
**Macromineral (g/kg EBW**^**0.75**^**)**				
Ca	Ca Ret. = − 0.0784 + 0.652 × Ca intake	0.74	0.034	< 0.001
P	P Ret. = − 0.0926 + 0.80 × P intake	0.86	0.021	< 0.001
Mg	^a^Mg Ret. = − 0.0065 + 0.113 × Mg intake	0.65	0.002	< 0.001
	^b^Mg Ret. = − 0.0065 + 0.079 × Mg intake	0.75	0.002	< 0.001
Na	^a^Na Ret. = − 0.0242 + 0.843 × Na intake	0.91	0.002	0.003
	^b^Na Ret. = − 0.0167 + 0.843 × Na intake	0.93	0.002	< 0.001
K	K Ret. = − 0.0379 + 0.590 × K intake	0.85	0.005	< 0.001
**Trace element (mg/kg EBW**^**0,75**^**)**				
Co	Co Ret. = − 0.0015 + 0.129 × Co intake	0.72	0.005	< 0.001
Cu	Cu Ret. = − 0.037 + 0.159 Cu intake	0.28	0.047	0.003
Mn	^a^Mn Ret. = 0.0138 + 0.0047 Mn intake	0.62	0.004	< 0.001
	^b^Mn Ret. = 0.0138 + 0.0077 Mn intake	0.79	0.003	< 0.001
Zn	Zn Ret. = 0.698 + 0.349 Zn intake	0.86	0.104	< 0.001
Cr	Cr Ret. = − 0.0055 + 0.804 Cr intake	0.25	0.013	0.01

*Ret* retained.

^a^Intact male; ^b^castrated male.

### Comparison of total dietary minerals requirements between the committees

**Figure 1:**
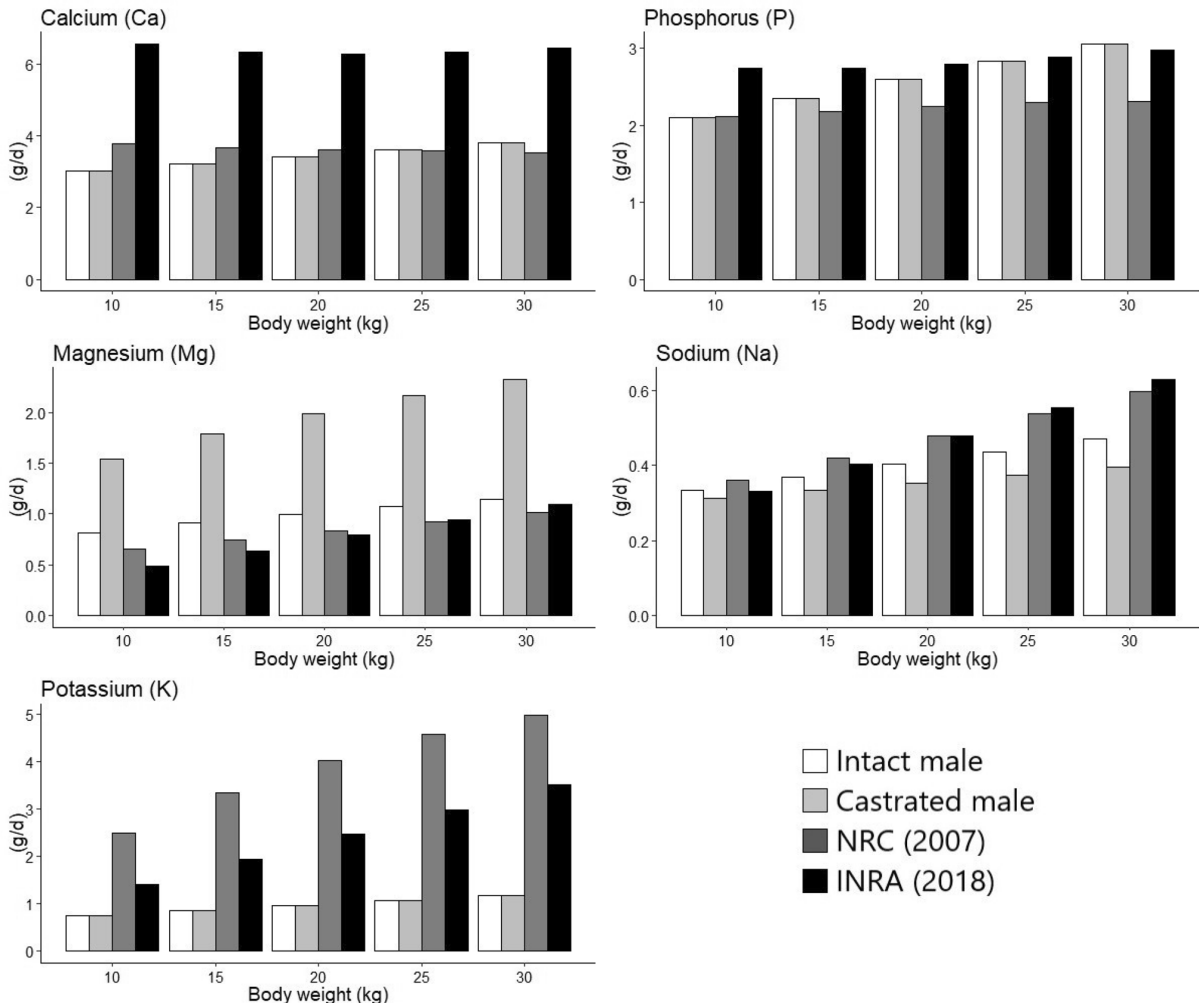
Comparison of total dietary macromineral requirements among the obtained results and the committees (INRA^6^ and NRC^3^).

**Figure 2:**
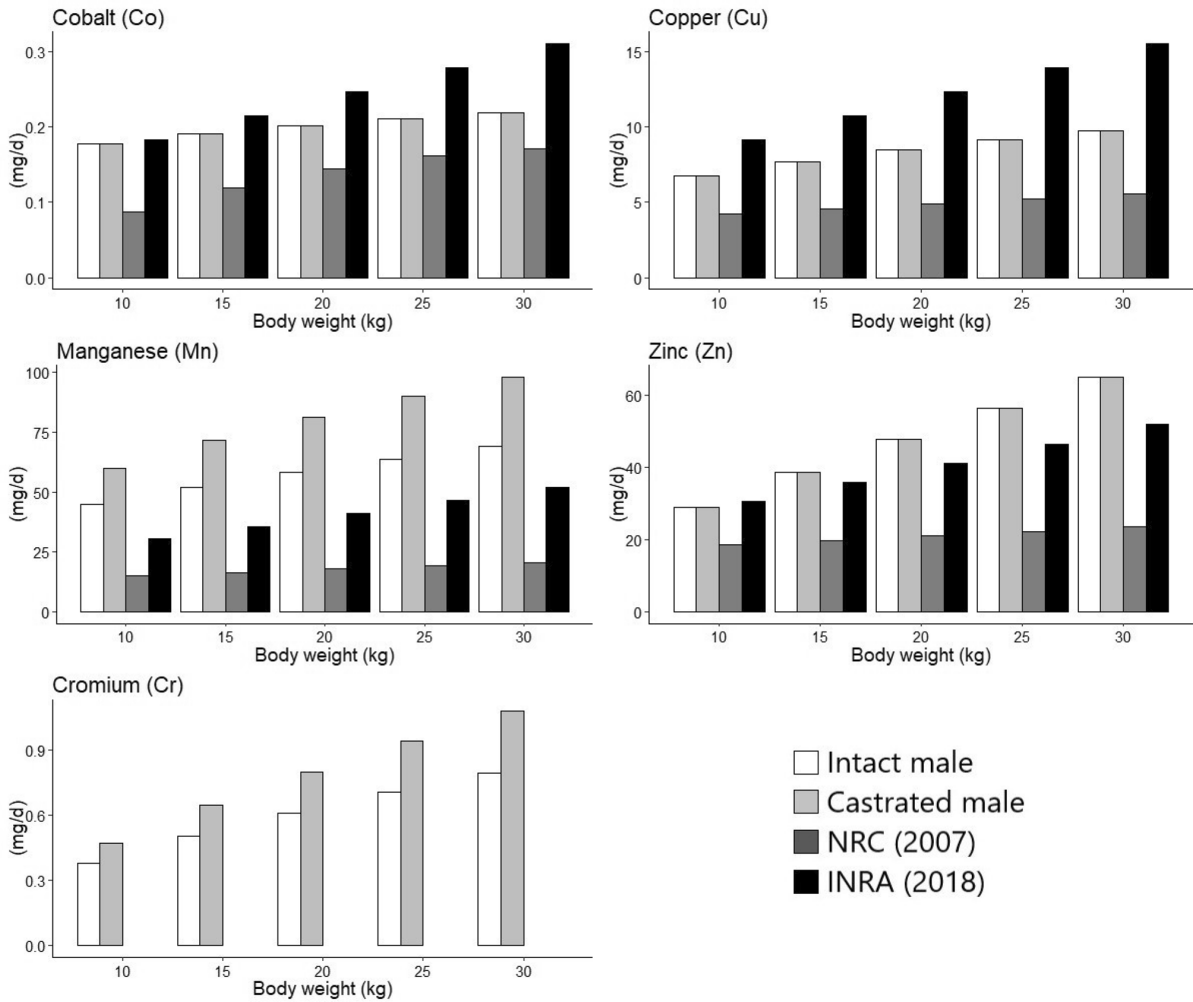
Comparison of total dietary trace mineral requirements among the obtained results and the committees (INRA^6^ and NRC^3^).

The total dietary requirements of this study and those presented at the NRC^3^ and INRA^6^ committees are shown in Figs. 1 and 2. The dietary requirements with 30 kg BW and an ADG of 200 g for Cu (9.77 mg/day), Mn (69.11 mg/day for IM and 98.03 mg/day for CM), and Co (0.21 mg/day) were higher than those proposed by the NRC^3^, which recommended 5.53, 20.53 and 0.17 mg/day for Cu, Mn and Co, respectively. However, the requirements of Co and Cu were lower than those reported by the INRA^6^ committee, which recommended 0.31 and 15.54 mg/day, respectively. Besides, the Zn level (64.99 mg/day) was higher than the values recommended (51.83 mg/day) by the INRA^6^ committee.

## Discussion

The NRC^3^, CSIRO^5^, and INRA^6^ international committees play an important role in nutritional recommendations for sheep, especially for those in the temperate region. In tropical scenarios, the nutritional requirements recommended by international committees may not be adequate to meet the physiological needs at different stages of animal life. Factors such as mathematical, environmental conditions^3^, genotype^16^, body composition, sex, and feed quality^7^ may influence the mineral requirements. In our study, the higher DM and mineral intake in sheep fed ad libitum resulted in greater performance (final BW, EBW, and ADG), indicating that the animals’ response was directly influenced by the level of intake. The DM intake is an important variable affecting animal performance because it guarantees the organism adequate nutrients and energy substrates for biochemical reactions to meet maintenance requirements. The 60% feed restriction was sufficient to meet the animals’ essential survival processes. Factors such as age, sex class, and genetic group influence nutritional requirements^5^.

The Ca is the most abundant mineral element in the animal body^6^, and approximately 98% percent of Ca in the body is located within the skeleton where it, along with phosphate anions, serves to provide structural strength and hardness to bone. Thus, lambs with a BW of 10 to 30 kg had decreased Ca concentration (% of EBW) and consequently net requirements of Ca. When compared to the committee’s recommendation for lambs weighing 20 kg and fed for an ADG of 200 g/day, the lambs had similar Ca requirements to those suggested by the NRC^3^. The mineral body composition oscillates according to the animal age and nutritional status. The bone tissue proportion will decrease because the largest amount of Ca and P in the body is in the bones^17^. Later, with the progression of growth, there is an increase in the proportion of fat followed by a reduction in the proportions of water, protein, and minerals in the body, particularly after 30 kg of BW. This pattern of nutrient deposition in the body is directly related to the stages of development of the bone, muscle, and adipose tissues^18^. Nevertheless, evaluating chemical constituents of Brazilian Somalis, in the same range of BW, there was a decrease in ash content in the animal's body, which leads to a reduction in Ca requirements^9^.

In the present study, the Na concentration remained constant as the BW increased (10 to 30 kg of BW). Most of the Na present in the animal body is located in the soft tissues and body fluids. A decrease in the water amount in the BW of the animals during this growth period was observed, which, in turn, consequently decreased the Na concentration in their bodies.

The sexual class is one of the factors that influence the chemical constituents of the animal's body^19,20^. However, the NRC^3^ and CSIRO^5^ have not provided inferences about the sex influence on mineral requirements. Differences in the requirements for Mg, Mn, and Cr were observed for Santa Ines males during the evaluated growth phase. Mg is closely associated with Ca and P. Approximately 70% of Mg in the total body is present in the skeleton. The Mg is an enzyme activator, including enzymes involved in the transfer of phosphate^21^. The CM have higher requirements for Mg, possibly due to the higher fat content in the body. Increases in the requirements of Mg and Cr for gain are directly related to the energy expenditure of lipogenesis.

Recent studies have shown that Cr affects carbohydrate and/or lipid metabolism^22,23^. The higher Cr gain net requirements for CM (0.84 mg/day vs. 0.62 mg/day for CM and IM, respectively) suggested that Cr in CM might be more active to increase glucose levels for muscle and adipose tissue. It is well established that Cr supplementation increases insulin responsiveness^24^, mainly in non-ruminants. The Cr is an element that stimulates lipogenesis and inhibits lipolysis.

The recommendations of different committees consider their calculations of endogenous losses from feces. The maintenance requirement for minerals involves not only urinary and fecal losses that occur, but also those that may occur by skin, sweat, and among others. Concerning the mineral retention coefficients, the values were lower for Mg, Na, and K, which may be related to the high amount of these minerals in the diet and consequently increased excretion. Thus, despite an improvement in the estimates, the retention coefficients found in this study were not definitive and may change as a function of tissue growth^25,26^. Animals at the beginning of life tend to have higher retention coefficients than adult animals. Therefore, the degree of variation in retention/absorption coefficients according to growth function is yet to be determined.

In our study, the Ca concentration (0.78 g/day) for the maintenance of hair sheep with a BW of 30 kg were lower than those recommended by the NRC and INRA (0.94 and 0.99 g/day, respectively). However, the P concentrations (0.98 g/day) for the maintenance were higher than the recommended by the NRC (0.73 g/day) and lower than the recommended by INRA (1.30 g/day). The requirement of Mg (0.03 g/day) was lower than those recommended by the NRC^3^ (0.09 g/day) and INRA^6^ (0.42 g/day). For Na, the maintenance requirements were 0.10 g/day for CM and 0.16 g/day for IM. These values were lower than those proposed by the NRC^3^ (0.32 g/day) and INRA^6^ (0.45 g/day). For K, the maintenance requirement was 0.30 g/day, which was lower than those suggested by the NRC^3^ (4.10 g/day) and INRA^6^ (3.15 g/day).

Trace elements, especially those more recently discovered, are required for maintaining the health and performance of farm animals^27,28^. Because these elements are either required in low concentrations or are commonly distributed in animal diets, deficiencies are likely to be exceptional under normal practical conditions.

Interestingly, this study demonstrated that sex affects the Mg and Mn requirements of males. Additionally, the results indicated that mineral requirements for weight gain and maintenance in Santa Ines sheep are different than those suggested by the global sheep feeding systems. It is essential for nutritionists and government regulators to establish the requirements for each mineral to optimize animal health and minimize tissue residues.

## Supplementary Information

Supplementary Information.
