# Supplementary material for: Macromineral and trace element requirements for Santa Ines sheep
Source: Sci Rep. 2021 Jun 10;11:12329. doi: 10.1038/s41598-021-91406-w (PMC8192910; doi:10.1038/s41598-021-91406-w)
Supplement: Supplementary file 1 — Supplementary Information. [file 41598_2021_91406_MOESM1_ESM.docx]

Macromineral and trace element requirements for Santa Ines sheep

# Dayanne Lima Sousa, Marcos Inácio Marcondes, Luciano Pinheiro Silva, Francisco Wellington Rodrigues Lima, Caio Julio Lima Herbster, Jocely Gomes Souza, João Paulo Pacheco Rodrigues, Leilson Rocha Bezerra, Ronaldo Lopes Oliveira, Elzania Sales Pereira.

**Supplementary Table**

| **Item** | **Intact male** | **Castrated male** | |
| --- | --- | --- | --- |
| BW (kg) | 13.64 | 15.75 | |
| EBW (kg) | 9.59 | 10.77 | |
| Water (%EBW) | 69.09 | 70.15 | |
| Protein (%EBW) | 16.15 | 15.65 | |
| Fat (%EBW) | 7.88 | 7.73 | |
| Ash (%EBW) | 6.38 | 6.35 | |
| Macrominerals (g/kg EBW) | | |  |
| Ca | 11.05 | 12.57 | |
| P | 8.66 | 9.68 | |
| Mg | 0.62 | 0.57 | |
| Na | 1.72 | 1.72 | |
| K | 2.30 | 2.23 | |
| Trace minerals (mg/kg EBW) | | |  |
| Co | 1.65 | 1.64 | |
| Cu | 5.40 | 6.63 | |
| Mn | 1.32 | 1.43 | |
| Zn | 36.89 | 42.24 | |
| Cr | 1.76 | 1.99 | |

**Table S1.** Body composition of baseline animals. BW = body weight; EBW = empty body weight.
